# “Neuropathological function estimations”: a user-friendly module for analyzing neural activity in neurological disorders

**DOI:** 10.1093/bioadv/vbaf083

**Published:** 2025-04-09

**Authors:** Alessia M Panait, Alex Kim, Rafael Rodriguez-Rojas, Lazaro M Sanchez-Rodriguez, Yasser Iturria-Medina

**Affiliations:** Enriched Health Sciences Program, Dawson College, Montreal, H3Z 1A4, Canada; Psychology Program, Dawson College, Montreal, H3Z 1A4, Canada; Faculty of Arts, McGill University, Montreal, H3A 0G4, Canada; HM CINAC (Centro Integral de Neurociencias Abarca Campal), Hospital Universitario HM Puerta del Sur, Mostoles. HM Hospitales, Madrid, 28015, Spain; Network Center for Biomedical Research on Neurodegenerative Diseases, Carlos III Institute, Madrid, 28029, Spain; Department of Neurology and Neurosurgery, McGill University, Montreal, H3A 2B4, Canada; McConnell Brain Imaging Centre, Montreal Neurological Institute, Montreal, H3A 2B4, Canada; Ludmer Centre for Neuroinformatics & Mental Health, McGill University, Montreal, H3A 2B4, Canada; Department of Neurology and Neurosurgery, McGill University, Montreal, H3A 2B4, Canada; McConnell Brain Imaging Centre, Montreal Neurological Institute, Montreal, H3A 2B4, Canada; Ludmer Centre for Neuroinformatics & Mental Health, McGill University, Montreal, H3A 2B4, Canada

## Abstract

**Motivation:**

This work introduces the Neuropathological Function Estimations software, designed to facilitate the study of neuronal activity alterations in neurological disorders without requiring programming expertise. With its user-friendly interface, researchers can input various data types to generate subject-specific functional brain models and decode neuropathological influences.

**Results:**

The software’s capabilities are validated through its application to Alzheimer’s disease, providing insights into neuronal excitability and disease mechanisms. This tool has the potential to enhance our understanding of the biological basis of *in vivo* neural activity and contribute to the development of personalized therapeutic interventions.

**Availability and implementation:**

The latest version of the software and support are freely available for noncommercial users through the Neuroinformatics for Personalized Medicine Lab (NeuroPM Lab) website at McGill University (https://www.neuropm-lab.com/neuropm-box.html). The software is maintained by the NeuroPM team. This publication is linked to version 1.0.

## 1 Introduction

Identifying the biological basis of neural activity abnormalities is instrumental for understanding brain disease (Sanchez-Rodriguez *et al.* 2024a,b). Since direct *in vivo* measurements of neuronal dysfunction often require invasive techniques, computational models have been developed as an alternative for studying neuronal activity in humans (Sanchez-Rodriguez *et al.* 2024a). Utilizing these models requires advanced programming and biophysical modeling expertise, however. Furthermore, while tools like The Virtual Brain (Schirner *et al.* 2022) serve as references for full-brain simulations, they currently lack built-in capabilities that can be widely applied to estimate biophysical alterations due to disease. To further democratize the characterization of causal pathological effects on neuronal activity in neurological disorders, we implemented a new module into the NeuroPM-box (Iturria-Medina *et al.* 2021). This user-friendly toolbox integrates diverse data to create realistic brain models and identify optimal personalized treatments.

## 2 Neuronal activity changes: core mechanisms in neurological disorders

Neural networks rely on balanced activity between excitatory and inhibitory neurons, which is essential for normal brain functioning. Disruptions in neuronal activity have been observed in many neurological disorders and are increasingly regarded as key mechanistic events (Ghatak *et al.* 2021). For instance, epileptogenesis often manifests as neuronal hyperexcitability and hypersynchrony (Chen *et al.* 2017). In Parkinson’s disease, dopamine depletion alters activity in the basal ganglia-thalamocortical network, which is responsible for the observed cardinal motor features (Foffani *et al.* 2019). In Alzheimer’s disease (AD), amyloid-beta (Aβ) plaques and tau tangles physically impair neurons, and their complex interactions seem to increase neuronal excitability, correlating with cognitive performance and several disease biomarkers (Sanchez-Rodriguez *et al.* 2024a).

We previously developed a biophysical framework to characterize neuronal activity alterations in AD (Sanchez-Rodriguez *et al.* 2024a). Aβ and tau accumulations in a brain region, observed in the subject’s PET scans, were assumed to linearly influence a neuronal excitability parameter, spreading across brain regions through connections identified via diffusion MRI. The model then simulated pathophysiological brain activity patterns and converted them into resting-state fMRI signals. Finally, the effects of Aβ and tau were quantified by fitting the generated signals to the subject’s real fMRI signals in terms of their fractional amplitude of low-frequency fluctuations (fALFF) (Jia *et al.* 2019), a reliable marker for neuronal activity (Supplementary Fig. S1). In this work, we extend the code applied to AD, rendering it more accessible, user-friendly, and easily adaptable for studying other conditions.

## 3 Software description

**Figure 1.**
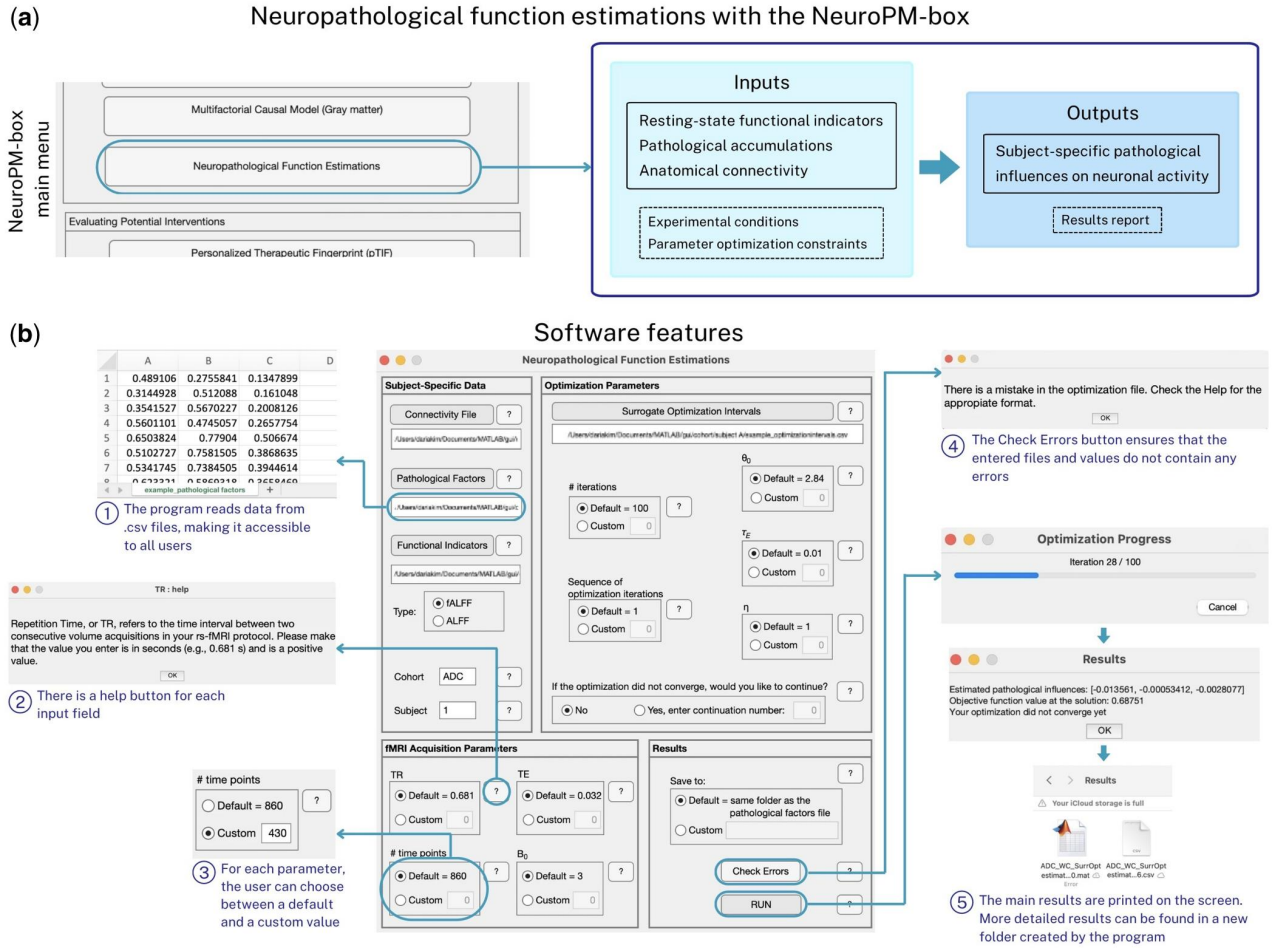
Functionalities of the Neuropathological Function Estimations module. (a) The software processes regional inputs including resting-state functional indicators and pathological accumulations, inter-regional anatomical connectivity, and experimental conditions/parameter optimization constraints. Consequently, the software identifies subject-specific pathological influences on neuronal activity and generates a comprehensive results report. (b) The tool is designed to be highly user-friendly. The main features that enhance the user experience have been highlighted in the figure. Default parameters and options are based on previous publications related to Alzheimer’s disease (Sanchez-Rodriguez *et al.* 2024a).

The software was designed to accelerate the investigation of neuronal activity alterations in neurological disorders. This graphical user interface, named “Neuropathological Function Estimations,” was developed in MATLAB R2024a (The MathWorks Inc., Natick, MA, USA). It is available as a standalone application for the major operating systems: Windows, macOS, and Linux, and requires the installation of MATLAB Runtime to function. The expected processing time varies depending on the operating system and device model, and on average it takes about an hour to complete 100 iterations of the optimization algorithm (see Supplementary Text S2: Instructions for installation and use). Researchers can upload their data in .csv format, which is widely accessible. The software requires four main inputs for each subject: (i) brain-wide distributions of the pathological factors included in the model, (ii) non-invasive *in vivo* indicators of functional alterations (typically obtained through resting-state fMRI), (iii) anatomical connectivity values for the regions defined in the user’s brain parcellation, and (iv) experimental conditions and parameter optimization constraints (see Fig. 1a and the exemplary simulated data provided with the software). Using this information, a whole-brain neuropathological influence model is generated within the code, and the most likely neuropathological influence parameters are identified through a surrogate optimization algorithm (https://www.mathworks.com/help/gads/surrogateopt.html).

The Neuropathological Function Estimations software features extensive personalization options and a clear layout, as shown in Fig. 1b. Each button group offers a default option with suggested standard values from the literature, as well as a custom option that allows users to specify their own parameters. Help texts are conveniently provided next to each parameter and button. Additionally, a complete step-by-step guide for utilizing the software, detailed installation instructions and mathematical formulations are included in the Supplementary Material. The software consists of several key sections. In the *Subject-Specific Data* section, users can input files containing biological measurements for each participant, select the type of functional indicators used, and tag their cohort and individual participants. Users can specify the parameters of their imaging protocol in the *fMRI Acquisition Parameters* section. The program will then generate resting-state fMRI signals according to these provided values. The *Optimization Parameters* section offers options regarding the optimization methods used to calculate results. For instance, users can choose the number of iterations for the run and decide whether to continue from a previous calculation that did not converge. Finally, the *Results* section includes options for selecting the file save location, as well as a “Check Errors” button to verify that the files and values entered do not contain any errors. Pressing the “RUN” button initiates the calculations. Once all calculations are complete, .mat and .csv files containing the outputs are generated and also appear in a pop-up window on the screen. The software has been tested on benchmark data, showing excellent alignment (average *R*^2^ = 0.97) between ground-truth and reconstructed neuronal activity parameter distributions (see Supplementary Fig. S2).

One significant limitation is that the current implementation is computationally intensive due to the simulations of the subject’s resting-state fMRI signals. However, acceleration can be achieved by reducing the number of time points used to calculate the functional indicators, without sacrificing biological reliability (Küblböck *et al.* 2014). Moreover, our model is based on the assumption that neuronal excitability can be reliably estimated by a simplified model of connected excitatory and inhibitory populations, which may pose a challenge in capturing the complexity of the living brain. Additionally, to ensure that the dynamical systems produce oscillatory behavior, it is recommended that users begin by applying narrow optimization intervals—see the discussion in (Sanchez-Rodriguez *et al.* 2024a) and the references therein for more information on the biophysical parameters of the model. The obtained values and comparisons among the subject-specific estimated pathophysiological influences help identify the molecular mechanisms underlying neuronal dysfunction and inform potential therapeutic interventions, among other possibilities (Sanchez-Rodriguez *et al.* 2024a,b).

## 4 Conclusion

The software reconstructs key biological quantities of interest and enhances our understanding of network reorganization patterns in the brain and their relationship with disease symptoms and prognosis. In the case of AD, we have validated the effectiveness of our computational approach in disease hypothesis testing (Sanchez-Rodriguez *et al.* 2024a,b), expanding the capabilities of the existing ecosystem of large-scale brain network simulations and parameter identification platforms. For example, the latest version of The Virtual Brain simulator (Schirner *et al.* 2022) enables Bayesian parameter optimization in epilepsy patient models. Our tool paves the way for new applications in other diseases that also cause neuronal activity alterations and complements the robust suite of methods for multifactorial disease analysis already available in the NeuroPM-box (Iturria-Medina *et al.* 2021). For instance, in Parkinson’s disease, dopamine transporter imaging techniques such as DaT 123I–FP-CIT scans and alpha-synuclein-specific PET (once available) can provide insights into the disease’s pathology, while fALFF and ALFF may serve as non-invasive, *in vivo* indicators of functional alterations (Lin *et al.* 2022, Khan *et al.* 2023, Sanchez-Rodriguez *et al.* 2024a). Future releases will offer greater flexibility in selecting affected parameters and neuronal activity markers, including additional resting-state fMRI or E/MEG features, as well as the ability to define different pathological influence equations. We also plan to add new features to handle increasing data volumes (e.g. finer brain parcellations requiring more physical memory to run the dynamical equations). An upcoming article describes an application that ensures scalability by using machine learning algorithms to predict functional indicators for any parameter combination from just a few grid simulations, reducing the need for ad-hoc runs. These ongoing efforts to refine the computational pipeline aim to improve both the efficiency and precision of the modeling approach. Understanding the mechanisms behind disease manifestations is crucial for developing personalized, effective therapeutic interventions.

## Supplementary Material

vbaf083_Supplementary_Data

## Data Availability

The latest version of the software and support are freely available for noncommercial users through the Neuroinformatics for Personalized Medicine Lab (NeuroPM Lab) website at McGill University (https://www.neuropm-lab.com/neuropm-box.html). The software is maintained by the NeuroPM team. This publication is linked to version 1.0.
