# Supplementary material for: “Neuropathological function estimations”: a user-friendly module for analyzing neural activity in neurological disorders
Source: Bioinform Adv. 2025 Apr 9;5(1):vbaf083. doi: 10.1093/bioadv/vbaf083 (PMC12017617; doi:10.1093/bioadv/vbaf083)
Supplement: vbaf083_Supplementary_Data [file vbaf083_supplementary_data.pdf]

Supplementary Information

'Neuropathological Function Estimations': A User-Friendly Module for Analyzing Neural Activity in Neurological Disorders

Alessia M. Panait[1*], Alex Kim[2,3*], Rafael Rodriguez-Rojas[4,5], Lazaro M. Sanchez-Rodriguez[6,7,8], Yasser Iturria-Medina[6,7,8]

[1]Enriched Health Sciences Program, Dawson College, Montreal, Canada.
[2]Psychology Program, Dawson College, Montreal, Canada.
[3]Faculty of Arts, McGill University, Montreal, Canada.
[4]HM CINAC (Centro Integral de Neurociencias Abarca Campal), Hospital Universitario HM Puerta del Sur, Mostoles. HM Hospitales, Madrid, Spain.
[5]Network Center for Biomedical Research on Neurodegenerative Diseases, Carlos III Institute, Madrid, Spain.
[6]Department of Neurology and Neurosurgery, McGill University, Montreal, Canada.
[7]McConnell Brain Imaging Centre, Montreal Neurological Institute, Montreal, Canada.
[8]Ludmer Centre for Neuroinformatics & Mental Health, Montreal, Canada.

*Contributed equally

Supplementary Text 1: A comprehensive step-by-step guide to utilizing the toolbox

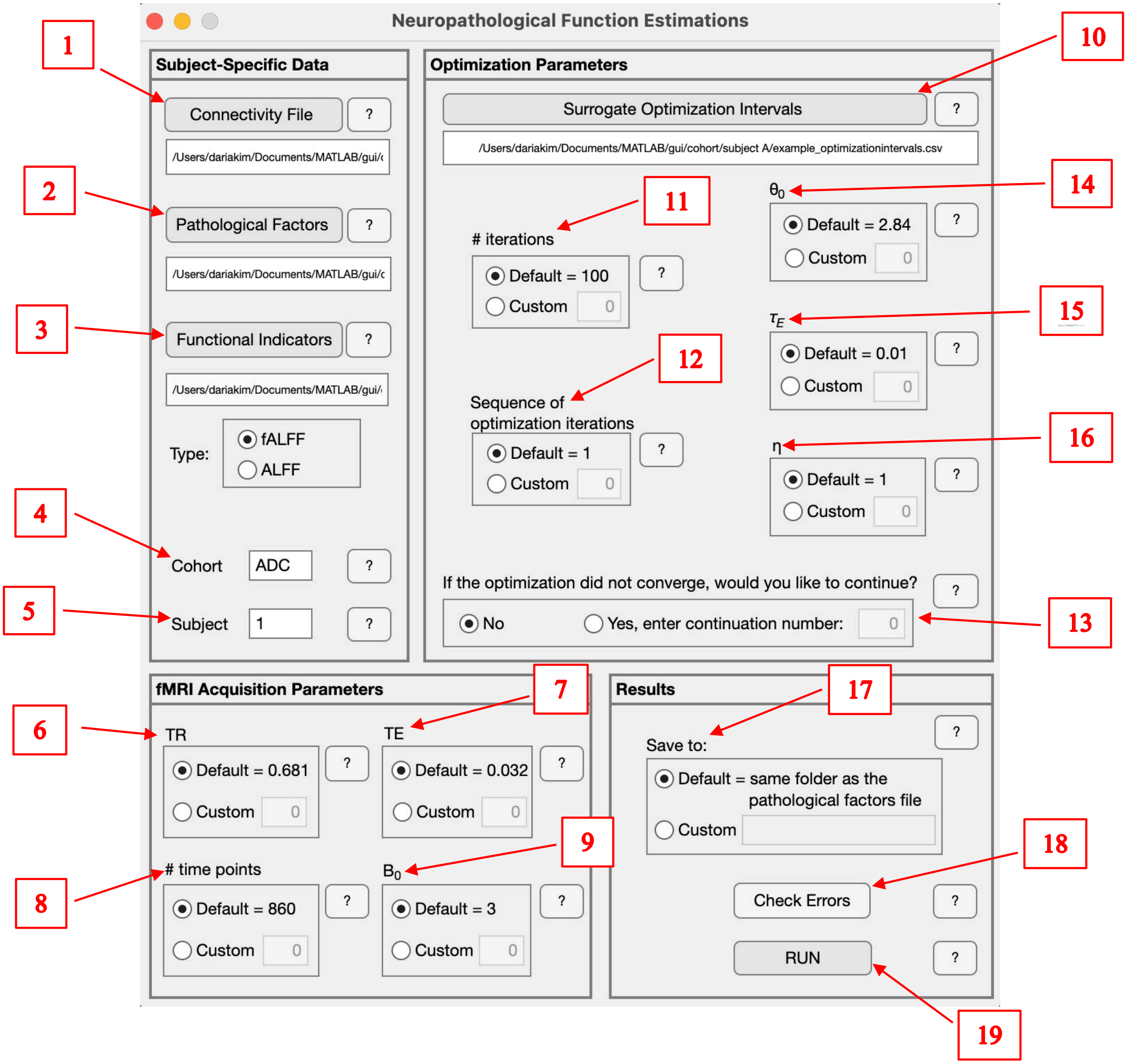

Legend:

<u>Subject-Specific Data</u>:

1) **Connectivity File**: table/matrix representing the connections between different brain regions. Each value in the matrix corresponds to the strength of a connection between two

regions and is typically in the [0,1] range. Please ensure the file you select is a .csv file. This input is required. Press on the button to enter the address to the file.

Example:

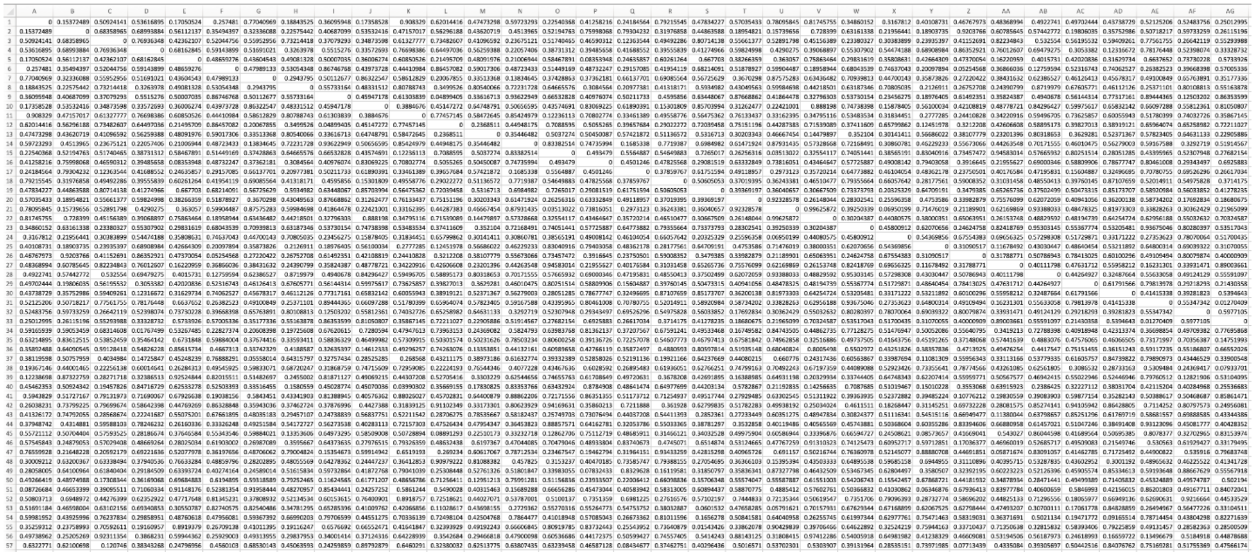

2) **Pathological Factors**: regional accumulation of disease-specific pathological agents (e.g., in Alzheimer's disease: Aβ, tau, and Aβ·tau imaged through PET). Please ensure that the file you select is a .csv file and that the columns refer to the number of factors and that the rows refer to the number of regions in your brain parcellation. Typically, each pathological factor is normalized to the [0,1] interval (by dividing by the maximum value across subjects and regions). This input is required. Press on the button to enter the address to the file.

Example:

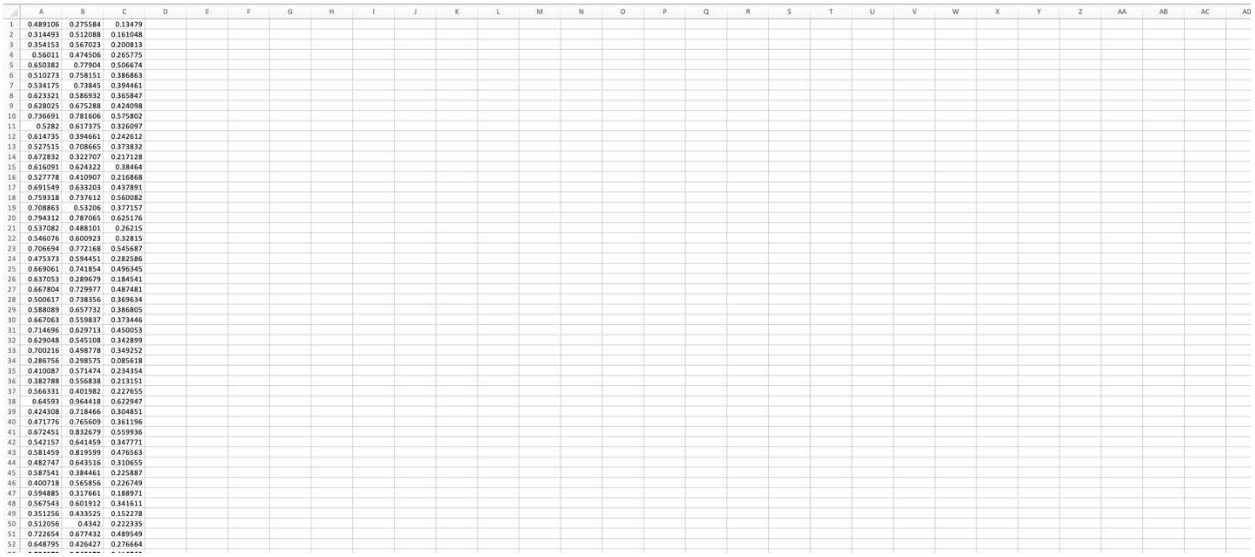

	A	B	C
1	0.489106	0.275584	0.13479
2	0.314493	0.512088	0.161048
3	0.354153	0.567023	0.200813
4	0.56011	0.474506	0.265775
5	0.650382	0.77904	0.506674
6	0.510273	0.758151	0.386863
7	0.534175	0.73845	0.394461
8	0.623321	0.586932	0.365847
9	0.628025	0.675288	0.424098
10	0.736691	0.781606	0.575802
11	0.5282	0.617375	0.326097
12	0.614735	0.394661	0.242612
13	0.527515	0.708665	0.373832
14	0.672832	0.322707	0.217128
15	0.616091	0.624322	0.38464
16	0.527778	0.410907	0.216868
17	0.691549	0.633203	0.437891
18	0.759318	0.737612	0.560082
19	0.708863	0.53206	0.377157
20	0.794312	0.787065	0.625176
21	0.537082	0.488101	0.26215
22	0.546076	0.600923	0.32815
23	0.706694	0.772168	0.545687
24	0.475373	0.594451	0.282586
25	0.669061	0.741854	0.496345
26	0.637053	0.289679	0.184541
27	0.667804	0.729977	0.487481
28	0.500617	0.738356	0.369634
29	0.588089	0.657732	0.386805
30	0.667063	0.559837	0.373446
31	0.714696	0.629713	0.450053
32	0.629048	0.545108	0.342899
33	0.700216	0.498778	0.349252
34	0.286756	0.298575	0.085618
35	0.410087	0.571474	0.234354
36	0.382788	0.556838	0.213151
37	0.566331	0.401982	0.227655
38	0.64593	0.964418	0.622947
39	0.424308	0.718466	0.304851
40	0.471776	0.765609	0.361196
41	0.672451	0.832679	0.559936
42	0.542157	0.641459	0.347771
43	0.581459	0.819599	0.476563
44	0.482747	0.643516	0.310655
45	0.587541	0.384461	0.225887
46	0.400718	0.565856	0.226749
47	0.594885	0.317661	0.188971
48	0.567543	0.601912	0.341611
49	0.351256	0.433525	0.152278
50	0.512056	0.4342	0.222335
51	0.722654	0.677432	0.489549
52	0.648795	0.426427	0.276664

3) **Functional Indicators**: measurements of brain activity. You can currently choose between two resting-state fMRI indicators: ALFF (Amplitude of Low-Frequency Fluctuations) or fALFF (fractional ALFF). Please ensure that the file you select is a .csv file with one column – each row containing a regional value – and then choose whether it refers to ALFF or fALFF. This input is required. Press on the button to enter the address to the file.

Example:

	A	B	C	D	E	F	G	H	I	J	K	L	M	N	O	P	Q	R	S	T	U	V	W	X	Y	Z	AA
1	0.1363236																										
2	0.0764621																										
3	0.0618722																										
4	0.0758781																										
5	0.1905681																										
6	0.1052917																										
7	0.1941309																										
8	0.1053589																										
9	0.2056265																										
10	0.0990135																										
11	0.1016689																										
12	0.0794165																										
13	0.1695747																										
14	0.1415749																										
15	0.1058301																										
16	0.199681																										
17	0.1342602																										
18	0.1548741																										
19	0.2266991																										
20	0.0929016																										
21	0.2077385																										
22	0.1829266																										
23	0.0928324																										
24	0.1421337																										
25	0.0441857																										
26	0.0586652																										
27	0.1381066																										
28	0.1859999																										
29	0.2157982																										
30	0.0633974																										
31	0.1519267																										
32	0.130341																										
33	0.03721																										
34	0.0995961																										
35	0.0698702																										
36	0.1765857																										
37	0.0804645																										
38	0.1389843																										
39	0.1016213																										
40	0.1605541																										
41	0.1024411																										
42	0.1640689																										
43	0.1609702																										
44	0.2282687																										
45	0.1350273																										
46	0.0702695																										
47	0.0863989																										
48	0.2360211																										

4) **Cohort**: tag for organizing the results on your group of subjects. This input is required.

5) **Subject**: participant’s identifier for organizing the results. This input is required.

<u>fMRI Acquisition Parameters</u>:

6) **TR (Repetition Time)**: time interval between two consecutive volume acquisitions in your rs-fMRI protocol. Please make sure that the value you enter is in seconds (e.g., 0.681 s) and is a positive value.

7) **TE (Time Echo)**: time interval between the RF pulse and the signal measurement. Please make sure that the value you enter is in seconds (e.g., 0.032 s) and is a positive value.

8) **# time points**: number of individual images (volumes) taken over time during a scan. Note that the entered value must be a positive number.

9) **B_0**: strength of the fMRI scanner’s main magnetic field, measured in Tesla (T). Note that the entered value must be a positive number (e.g., 3 T or 1.5 T).

Optimization Parameters:

10) **Surrogate Optimization Intervals**: a .csv file containing surrogate optimization options such as the bounds and constraints. This input is required. Press on the button to enter the address to the file. The file should be organized as follows:

    First column: lower bounds (lb), i.e., a lower constraint for the interval in which each factor's neuronal activity influences are searched for.

    Second column: upper bounds (ub), i.e., an upper constraint for the interval in which each factor's neuronal activity influences are searched for.

    Next N-factor columns [optional]: linear inequality matrix for establishing the relationships between the neuronal activity influences.

    Last column [optional]: linear inequality constraints for the neuronal activity influences.

    The program will search for a global minimum of the objective function in the region lb <= x <= ub, that satisfies the inequalities A*x <= b. For more details, see https://www.mathworks.com/help/gads/surrogateopt.html

    Example:

	A	B	C	D	E	F	G	H	I	J	K	L	M	N	O	P
1	-0.02	0.02	1	1	1	0.02										
2	-0.02	0.02	-1	-1	-1	0.02										
3	-0.02	0.02	1	1	-1	0.02										
4	NaN	NaN	1	-1	-1	0.02										
5	NaN	NaN	1	-1	1	0.02										
6	NaN	NaN	-1	1	1	0.02										
7	NaN	NaN	-1	1	-1	0.02										
8	NaN	NaN	-1	-1	1	0.02										
9																
10																
11																
12																
13																
14																
15																
16																
17																
18																
19																
20																
21																
22																
23																
24																
25																
26																
27																
28																

11) **# iterations**: determines how many times the optimization algorithm will update the variables to improve the solution. A higher number of iterations makes it more likely for the optimization to converge, however it may take longer to complete. A lower number may produce results faster but could stop before finding an optimal solution. Note that the entered value must be a positive number.

12) **Sequence of optimization iterations**: creates an initial seed for the optimization, for reproducibility, and a tag for saving the results. We recommend exploring multiple sequences of random sample points to retain the best solution in terms of the objective function value as the optimization algorithm, surrogateopt, will perform different evaluations based on the seed value. Note that the entered value must be a positive number.

13) **Continuation number**: the trial from which you would like to resume the calculations. For example, if you ran the calculations once and the optimization did not converge, you would enter "1" in the textbox to pick up from the initial ("0") trial. The "Continuation" section is used if the optimization fails to converge at the initially entered number of iterations. The algorithm will start from the previously evaluated points Note that the entered value must be a positive integer.

14) $\boldsymbol{\theta_0}$: baseline neuronal firing parameter. Neuropathological alterations are modeled as linear deviations from this value by each pathological factor. Note that the entered value must be a positive number.

15) $\boldsymbol{\tau_E}$: time-constant controlling the decay of excitatory activity after stimulation (i.e., frequency of the neuronal oscillators). The inhibitory analogous parameter is twice the selected value for τ_E. Please make sure that the value you enter is in seconds (e.g., 0.01 s) and is a positive value.

16) $\boldsymbol{\eta}$: global coupling strength scaling the anatomical connectivity matrix. Note that the entered value must be a positive number.

<u>Results</u>:

17) **Save to**: allows you to select the location where the results files will be saved. If you click on "Default", the results will be saved in the same location as your "Pathological Factors" file. If you click on "Custom", a window will open, allowing you to navigate through your folders and choose a specific location.

18) **Check Errors**: verifies that the files and values you have entered do not contain any errors. If an error is found, a window will open with an explanation of the issue, allowing you to correct it before proceeding. It is strongly recommended to click this button and review any errors before starting each trial.

19) **RUN**: The “Run” button starts the calculations once you have entered all required files and values and (optionally) checked for errors. After clicking this button, a progress bar will appear, showing the number of completed iterations. You may cancel the optimization at any point. The program will finish the current iteration and save the evaluations up to that point. The main results are printed on the screen.

<u>Outputs</u>:

The program will create two files for each sequence of optimization iterations:

a) A .mat file (tagged by cohort, subject, sequence of optimization iterations and continuation number) with the following variables:

   ExcitabilityEffect: a [1 x number of factors] vector with the estimated pathological factors influences on neuronal activity.

   ObjFuncVal: the objective function value at the estimated pathological influences.

   exitflag: the reason why the optimization algorithm stopped.

   output: a structure describing the optimization procedure

   trials: a structure containing all the evaluated points and the objective function values at those points.

   For more information, please see <u>https://www.mathworks.com/help/gads/surrogateopt.html</u>.

b) A .csv file with the summary results at the last run trial of the optimization procedure. For example:

ExcitabilityEffect_Factor1	ExcitabilityEffect_Factor2	ExcitabilityEffect_Factor3	ObjFuncVal	exitflag
-0.013560923	-0.000534117	-0.002807747	0.687509721	0

Supplementary Text 2: Instructions for installation and use

System requirements:

⇒ The software has been tested on MATLAB (versions: R2021b, R2023b, R2024a) and as a standalone application on Windows, Linux, and macOS.

⇒ MATLAB Runtime is required to run the GUI (see **Installation guide**).

Installation guide:

Instructions to install:

⇒ **For Windows**:

1) Navigate to the folder where the App Installer is located.
2) Double-click on MyAppInstaller_web.exe.
3) Choose the locations where to install the software and MATLAB Runtime and follow the on-screen instructions.

⇒ **For macOS**:

1) Navigate to the folder where the App Installer is located.
2) Run the App Installer:
   - Open the terminal and execute the following command:
     `open /path/to/your/appinstaller/folder/MyAppInstaller_mcr.app`
3) Choose the locations where to install the software and MATLAB Runtime.

Note: some mac systems may deny external applications to run. In that case, please follow these steps:

1) Open the Terminal or navigate to the folder where the App Installer is located.
2) Execute the following command:
   `chmod –R 755 /path/to/your/appinstaller`
   - In your path, if there are any spaces in the folder names, replace the spaces with (\ ).
3) Double-click on MyAppInstaller_mcr:
   - A prompt will appear saying the app cannot be opened because the developer is not verified.
   - Go to Settings > Privacy & Security. Find the message about the app and click Open Anyway to allow it to launch.

- A prompt will appear asking if you are sure you want to open the app despite the developer not being verified.
- Select Open Anyway to launch the app.

4) Choose the locations and download MATLAB Runtime and the software.

⇒ **For Linux**:

1) Navigate to the folder where the App Installer is located.
2) Double-click on MyAppInstaller_web.install.
3) Choose the locations where to install the software and MATLAB Runtime and follow the on-screen instructions.

Typical install time:

⇒ The typical installation time on a "normal" desktop computer ranges from **5 to 15 minutes**, depending on system performance and available resources.

Demo:

Instructions to run on data:

⇒ **For Windows**:

1) Navigate to the directory where the software is located.
2) Run the application:
   - Double-click neuropathological_function_estimations.exe to launch the software.

⇒ **For macOS**:

1) Navigate to the folder where the software is located.
2) Run the application:
   - Open a terminal and execute the following command to launch the software.

```
open /path/to/your/app/folder/neuropathological_function_estimations.app
```

⇒ **For Linux**:

1) Navigate to the folder where the software is located.
2) Run the application:
   - Open a terminal and execute the following command to launch the software.

```
./run_neuropathological_function_estimations.sh
/path/to/your/runtime/folder
```

For illustrative examples, please refer to **Supplementary Text 1: A comprehensive step-by-step guide to utilizing the toolbox** along with the example data provided with the software.

How to run the software on new data:

⇒ When you open the software, you will be prompted to upload four files: connectivity, pathological factors, functional indicators, and surrogate optimization intervals (please check that the format of files aligns with the provided examples). Then, you will be asked to enter the cohort and the subject, as well as the required values. Note that you can always modify the parameters and values by choosing to enter a custom value. Lastly, you will be invited to choose the location where you would like to save your results, (optionally) check for errors and press RUN to start the calculations. For more information, please see **Supplementary Text 1: A comprehensive step-by-step guide to utilizing the toolbox.**

Expected output:

⇒ The software opens a window with a summary of results, containing estimated pathological influences, objective function value at the solution, and the reason why the optimization algorithm stopped. It also creates two files: a .mat file and .csv file that contain all the information about your parameter estimations results. For more information, please see **Supplementary Text 1: A comprehensive step-by-step guide to utilizing the toolbox.**

Expected run time for demo on a "normal" desktop computer:

⇒ Here is the expected run time based on trials with example data and default parameters:

- **Windows**: On Intel64 Family 6 Model 186 Stepping 2 GenuineIntel @ 2.2 GHz, 16 GB Total Physical Memory: ~1h/100 iterations
- **macOS**: On Apple M2, @ 3.5 GHz, 8 GB Total Physical Memory: ~1h20min/100 iterations
- **Linux**: On Intel(R) Core(TM) i7-9700K CPU @ 3.6 GHz, 32 GB Total Physical Memory: ~ 30min/100 iterations

Supplementary Text 3: Personalized neuronal activity model

For each participant, the regional BOLD signals are generated through different transformations in the biophysical model (Sanchez-Rodriguez, Ahmed F Khan, *et al.*, 2024; Sanchez-Rodriguez, Gleb Bezgin, *et al.*, 2024a). Firstly, the excitatory and inhibitory firing rates in neural mass k, $E_k(t)$ and $I_k(t)$, are obtained from the following coupled differential equations:

$$\dot{E}_k = \frac{1}{\tau_E}\left[-E_k + S\left(x_{E,k}\right)\right]$$

$$\dot{I}_k = \frac{1}{\tau_I}\left[-I_k + S\left(x_{I,k}\right)\right]$$

$$x_{E,k} = C_{EE}E_k - C_{IE}I_k + P + \frac{\eta}{N}\sum_{l=1,l\neq k}^{N} \mathrm{C}_{lk}E_l$$

$$x_{I,k} = C_{EI}E_k - C_{II}I_k$$

where $S_I\left(x_{I,k}\right) = \frac{1}{1+\exp\left[-a_I\left(x_{I,k}-\theta_I\right)\right]} - \frac{1}{1+\exp\left[a_I\theta_I\right]}$ and $S_E\left(x_{E,k}\right) = \frac{1}{1+\exp\left[-a_E\left(x_{E,k}-\theta_{E,k}\right)\right]} - \frac{1}{1+\exp\left[a_E\theta_{E,k}\right]}$ are sigmoidal activation functions of the input currents $x_{E,k}$ and $x_{I,k}$ (Wilson and Cowan, 1972; Daffertshofer and van Wijk, 2011; Gjorgjieva *et al.*, 2016). The model's dynamics is modulated by a global scaling factor, η/N , operating on the cortico-cortical connections, C, where η represents coupling strength and N is the total number of modeled brain regions. Additionally, the firing threshold parameters, θ, determine the firing properties of the neuronal populations in relation to the local input currents. When θ is lower than a baseline value, neuronal populations become hyperexcitable, meaning they respond to lower levels of input current with increased firing rate (van Nifterick *et al.*, 2022; Daffertshofer and van Wijk, 2011). Conversely, setting θ higher than the baseline induces hypoexcitability in neuronal populations.

We assume that the effective regional firing threshold values are linear fluctuations from a normal baseline value θ_0 due to the considered pathophysiological factors:

$$\theta_k = \theta_0 + \sum_{l=1}^{N_{factors}} \theta_E^{Factor_l} \cdot Factor_{l,k}$$

Based on the much larger excitatory prevalence in the cortex (Maestú *et al.*, 2021; Lauterborn *et al.*, 2021), we quantify the regional activity profiles through the excitatory firing threshold θ_E, while the inhibitory variables are coupled via the above system. For instance, in the Alzheimer's

disease model (Sanchez-Rodriguez, Ahmed F Khan, *et al.*, 2024; Sanchez-Rodriguez, Gleb Bezgin, *et al.*, 2024a), the firing parameter is mediated by the local amyloid-beta ($A\beta$) and tau loads along with their synergistic interaction: $\theta_{E,k} = \theta_0 + \theta_E^{A\beta} \cdot A\beta_k + \theta_E^{Tau} \cdot Tau_k + \theta_E^{A\beta \cdot Tau} \cdot A\beta_k \cdot Tau_k$.

The regional BOLD signal relates to the action potential arriving at the neuronal populations (Logothetis *et al.*, 2001; Sotero and Trujillo-Barreto, 2008; Valdes-Sosa *et al.*, 2009). All quantities are normalized to baseline values: $\xi_{E,k} = \frac{s_{E,k}}{{s_{E,k}}^0}$ (normalized excitatory input) and $\xi_{I,k} = \frac{s_{I,k}}{{s_{I,k}}^0}$ (normalized inhibitory input), where the superscript denotes values at rest.

Changes in glucose consumption ($g_{E,k}$ and $g_{I,k}$) are linked to the excitatory and inhibitory neuronal inputs in region k. The glucose variables transform into metabolic rates of oxygen for excitatory ($m_{E,k}$) and inhibitory ($m_{I,k}$) activities, and total oxygen consumption (m_k):

$$\dot{g}_{E,k} = z_{E,k}$$

$$\dot{z}_{E,k} = \frac{-2}{\varkappa_E} z_{E,k} - \frac{1}{{\varkappa_E}^2}\left(g_{E,k} - 1\right) + \frac{h_E}{\varkappa_E}\left(\xi_{E,k} - 1\right)$$

$$\dot{g}_{I,k} = z_{I,k}$$

$$\dot{z}_{I,k} = \frac{-2}{\varkappa_I} z_{I,k} - \frac{1}{{\varkappa_I}^2}\left(g_{I,k} - 1\right) + \frac{h_I}{\varkappa_I}\left(\xi_{I,k} - 1\right)$$

$$m_{E,k}(t) = \frac{2 - x(t)}{2 - x_0} g_{E,k}(t)$$

$$m_{I,k}(t) = g_{I,k}(t)$$

$$m_k(t) = \frac{\gamma m_{E,k}(t) + m_{I,k}(t)}{\gamma + 1}$$

$$x(t) = \frac{1}{1 + exp\left[c\left(d - g_{E,k}(t)\right)\right]}$$

Cerebral blood flow (f_k) is modeled as follows (Friston *et al.*, 2000), assuming that CBF is coupled to the excitatory activity:

$$\dot{f}_k = y_k$$

$$\dot{y}_k = \frac{-2}{\varkappa_f} y_k - \frac{1}{{\varkappa_f}^2}(f_k - 1) + \mu\left(\xi_{E,k} - 1\right)$$

The outputs of the metabolic and vascular modules are converted to normalized cerebral blood volume (b_k) and deoxy-hemoglobin (q_k) content through the Balloon model (Buxton *et al.*, 1998):

$$\dot{b}_k = \frac{1}{\varkappa_0}(f_k - f_{out})$$

$$\dot{q}_k = \frac{1}{\varkappa_0}\left(m_k - f_{out}\frac{q_k}{b_k}\right)$$

$$f_{out} = b_k^{\frac{1}{\zeta}}$$

The regional BOLD signal is finally obtained by using the following linear observation equation:

$$BOLD_k(t) = V_0\big(a_1(1 - q_k) - a_2(1 - b_k)\big)$$

where $a_1 = 4.3\Upsilon_0 E_0 \cdot TE + \varepsilon r_0 E_0 \cdot TE$ and $a_2 = \varepsilon r_0 E_0 \cdot TE + \varepsilon - 1$ are parameters that depend on the experimental conditions (field strength, TE) (Obata *et al.*, 2004; Simon and Buxton, 2015; Archila-Meléndez *et al.*, 2020; Deco *et al.*, 2018).

Supplementary Text 3—Table 1. Dynamical model parameters

Parameter	Definition	Value	Ref.
$\begin{bmatrix} E_0 \\ I_0 \\ g_{E0} \\ z_{E0} \\ g_{I0} \\ z_{I0} \\ f_0 \\ y_0 \\ b_0 \\ q_0 \end{bmatrix}$	Initial conditions	$\begin{bmatrix} 0.075 \\ 0.01 \\ 1 \\ 0 \\ 1 \\ 0 \\ 1 \\ 0 \\ 1 \\ 1 \end{bmatrix}$	(Sotero and Trujillo-Barreto, 2007, 2008; Sotero *et al.*, 2009; Valdes-Sosa *et al.*, 2009)
τ_I	Time-constant controlling the decay of inhibitory activity after stimulation	$2 \cdot \tau_E$	(Abeysuriya *et al.*, 2018)
τ_E	Time-constant controlling the decay of excitatory activity after stimulation	depends on the experiment (Default: 0.01 s)	(Abeysuriya *et al.*, 2018)
C_{II}	Local inhibitory-inhibitory connection strength	1.2	(Gjorgjieva *et al.*, 2016; Meijer *et al.*, 2015; Wilson and Cowan, 1972)
C_{EI}	Local excitatory-inhibitory connection strength	6	(Gjorgjieva *et al.*, 2016; Meijer *et al.*, 2015; Wilson and Cowan, 1972)
C_{EE}	Local excitatory-excitatory connection strength	6.4	(Gjorgjieva *et al.*, 2016; Meijer *et al.*, 2015; Wilson and Cowan, 1972)
C_{IE}	Local inhibitory-excitatory connection strength	4.8	(Gjorgjieva *et al.*, 2016; Meijer *et al.*, 2015; Wilson and Cowan, 1972)
P	Average constant external input received by the excitatory population	0.65	(Gjorgjieva *et al.*, 2016; Meijer *et al.*, 2015; Wilson and Cowan, 1972)
a_I	Maximum slope of the inhibitory sigmoidal activation function	1	(Abeysuriya *et al.*, 2018)
a_E	Maximum slope of the excitatory sigmoidal activation function	1	(Abeysuriya *et al.*, 2018)
θ_{I_0}	Position of the inhibitory sigmoidal firing function's threshold for activation	4	(Gjorgjieva *et al.*, 2016; Meijer *et al.*, 2015; Wilson and Cowan, 1972)
θ_{E_0}	Position of the excitatory sigmoidal firing function's threshold for activation	depends on the experiment (Default: 2.84)	(Sanchez-Rodriguez, Ahmed F Khan, *et al.*, 2024; Sanchez-Rodriguez, Gleb Bezgin, *et al.*, 2024a)

η	Global coupling strength scaling the anatomical connectivity matrix C_{lk}	depends on the experiment (Default: 1)	(Gjorgjieva *et al.*, 2016; Meijer *et al.*, 2015; Wilson and Cowan, 1972; Daffertshofer and van Wijk, 2011; Abeysuriya *et al.*, 2018)
N	Number of brain regions of interest	depends on the experiment	(Sanchez-Rodriguez, Ahmed F Khan, *et al.*, 2024; Sanchez-Rodriguez, Gleb Bezgin, *et al.*, 2024a)
h_E	Efficacy of glucose consumption response to excitation	1	(Sotero and Trujillo-Barreto, 2007, 2008; Sotero *et al.*, 2009; Valdes-Sosa *et al.*, 2009)
h_I	Efficacy of glucose consumption response to inhibition	1	(Sotero and Trujillo-Barreto, 2007, 2008; Sotero *et al.*, 2009; Valdes-Sosa *et al.*, 2009)
$\varkappa_E$	Time-constant of the excitatory glucose consumption impulse response.	1 s	(Sotero and Trujillo-Barreto, 2007, 2008; Sotero *et al.*, 2009; Valdes-Sosa *et al.*, 2009)
$\varkappa_I$	Time-constant of the inhibitory glucose consumption impulse response.	1 s	(Sotero and Trujillo-Barreto, 2007, 2008; Sotero *et al.*, 2009; Valdes-Sosa *et al.*, 2009)
c	Steepness of the sigmoid function x	2.5	(Sotero and Trujillo-Barreto, 2007, 2008; Sotero *et al.*, 2009; Valdes-Sosa *et al.*, 2009)
d	Position of the threshold of the sigmoid function x	1.6	(Sotero and Trujillo-Barreto, 2007, 2008; Sotero *et al.*, 2009; Valdes-Sosa *et al.*, 2009)
γ	Baseline ratio of excitatory to inhibitory synaptic activity in the voxel	5	(Sotero and Trujillo-Barreto, 2007, 2008; Sotero *et al.*, 2009; Valdes-Sosa *et al.*, 2009)
x_0	Fraction of glucose following the glycogenolitic pathway at rest	$\frac{1}{1+exp\left[c\left(d-1(t)\right)\right]}$	(Sotero and Trujillo-Barreto, 2007, 2008; Sotero *et al.*, 2009; Valdes-Sosa *et al.*, 2009)
μ	Efficacy of blood flow response to excitation	0.8	(Sotero and Trujillo-Barreto, 2007, 2008; Sotero *et al.*, 2009; Valdes-Sosa *et al.*, 2009)
$\varkappa_f$	Time constant for CBF response	1.7	(Sotero and Trujillo-Barreto, 2007, 2008; Sotero *et al.*, 2009; Valdes-Sosa *et al.*, 2009)
$\varkappa_0$	Transit time through the balloon	1	(Sotero and Trujillo-Barreto, 2007, 2008; Sotero *et al.*, 2009; Valdes-Sosa *et al.*, 2009)

ζ	Coefficient of the steady state flow-volume relationship	0.4	(Sotero and Trujillo-Barreto, 2007, 2008; Sotero *et al.*, 2009; Valdes-Sosa *et al.*, 2009)
V_0	Baseline blood volume	0.03	(Sotero and Trujillo-Barreto, 2007, 2008; Sotero *et al.*, 2009; Valdes-Sosa *et al.*, 2009)
E_0	Baseline oxygen extraction fraction	0.4	(Obata *et al.*, 2004; Simon and Buxton, 2015; Archila-Meléndez *et al.*, 2020)
Υ_0	frequency offset of a fully deoxygenated blood vessel	80.6 s^{-1} at 3 T<br>40.3 s^{-1} at 1.5 T	(Obata *et al.*, 2004; Simon and Buxton, 2015; Archila-Meléndez *et al.*, 2020)
r_0	Slope defining the dependence of the R2* relaxation rate on blood oxygenation	178 s^{-1} at 3 T<br>25 s^{-1} at 1.5 T	(Obata *et al.*, 2004; Simon and Buxton, 2015; Archila-Meléndez *et al.*, 2020)
ε	Intrinsic ratio of blood to tissue signals at rest	0.24 at 3 T<br>1.43 at 1.5 T	(Obata *et al.*, 2004; Simon and Buxton, 2015; Archila-Meléndez *et al.*, 2020)
TE	Echo time	depends on the experiment (Default: 0.032 s)	(Sanchez-Rodriguez, Ahmed F Khan, *et al.*, 2024; Sanchez-Rodriguez, Gleb Bezgin, *et al.*, 2024a)
TR	Time to repeat	depends on the experiment (Default: 3 s)	(Sanchez-Rodriguez, Ahmed F Khan, *et al.*, 2024; Sanchez-Rodriguez, Gleb Bezgin, *et al.*, 2024a)
#time points	Number of volumes acquired	depends on the experiment (Default: 860)	(Sanchez-Rodriguez, Ahmed F Khan, *et al.*, 2024; Sanchez-Rodriguez, Gleb Bezgin, *et al.*, 2024a)

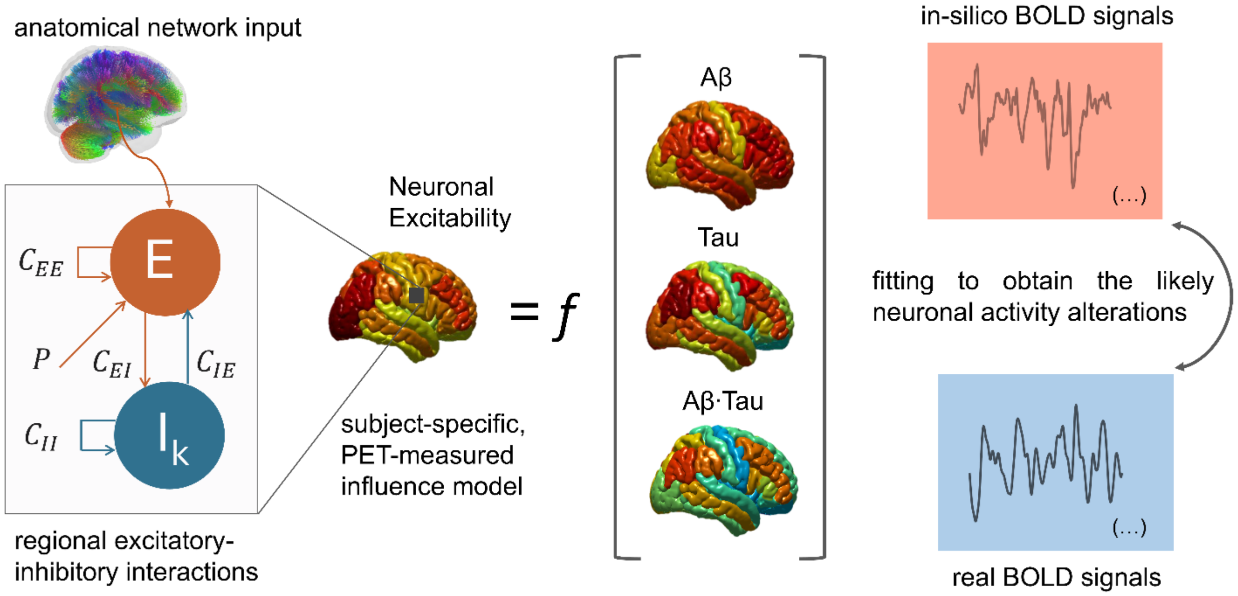

Supplementary Figure 1. Alzheimer's neuropathological influence model.

It is assumed that the functional alterations caused by accumulations of amyloid beta (Aβ) plaques and tau neurofibrillary tangles (Tau) spread across brain regions through connections identified via diffusion MRI. The model simulates brain activity patterns and converts them into resting-state fMRI signals. The effects of Aβ and Tau, and their synergistic interaction, are quantified by fitting the in-silico BOLD signals to real BOLD signals in terms of their fractional amplitude of low-frequency fluctuations (fALFF) (Sanchez-Rodriguez,L.M., Khan,Ahmed F, et al., 2024; Sanchez-Rodriguez,L.M., Bezgin G, et al., 2024b).

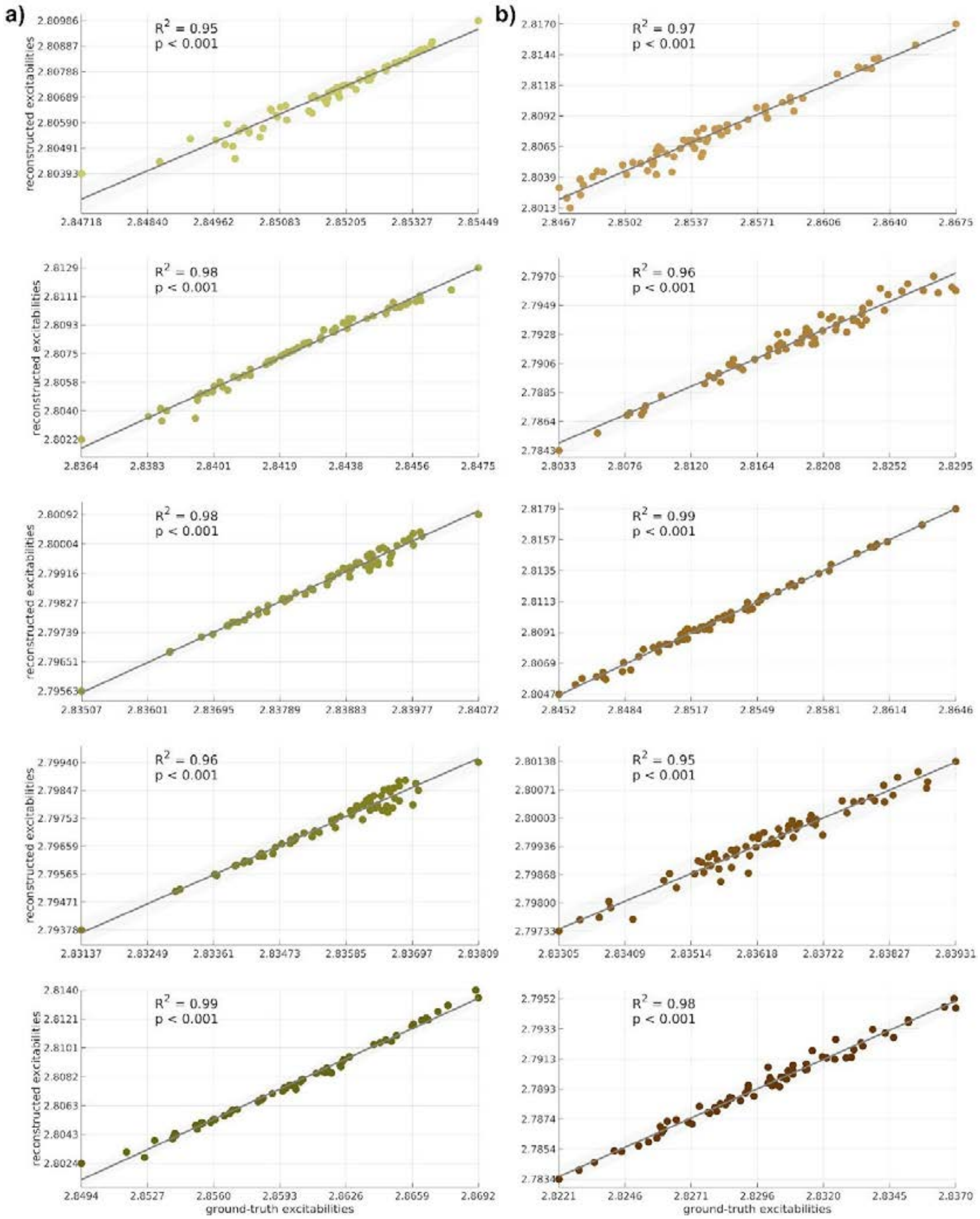

Supplementary Figure 2. The software's performance on ground-truth benchmarks.

a) Comparison of neuronal excitabilities reconstructed with the software and their ground-truth values, for the pathological factor distributions provided in the exemplary dataset. b) Similar to (a), but showing five randomly generated dummy datasets with a new set of pathological factor distributions. The coefficients of determination and p-values are displayed at the top of each panel. The error bands denote 95% confidence intervals.

References

Abeysuriya,R.G. *et al.* (2018) A biophysical model of dynamic balancing of excitation and inhibition in fast oscillatory large-scale networks.

Archila-Meléndez,M.E. *et al.* (2020) Modeling the impact of neurovascular coupling impairments on BOLD-based functional connectivity at rest. *Neuroimage*, **218**.

Buxton,R.B. *et al.* (1998) Dynamics of blood flow and oxygenation changes during brain activation: The balloon model. *Magn Reson Med*, **39**, 855–864.

Daffertshofer,A. and van Wijk,B.C.M. (2011) On the Influence of Amplitude on the Connectivity between Phases. *Front Neuroinform*, **5**, 6.

Deco,G. *et al.* (2018) Whole-Brain Multimodal Neuroimaging Model Using Serotonin Receptor Maps Explains Non-linear Functional Effects of LSD. *Current Biology*, **28**, 3065-3074.e6.

Friston,K.J. *et al.* (2000) Nonlinear responses in fMRI: The balloon model, Volterra kernels, and other hemodynamics. *Neuroimage*, **12**, 466–477.

Gjorgjieva,J. *et al.* (2016) Homeostatic activity-dependent tuning of recurrent networks for robust propagation of activity. *Journal of Neuroscience*, **36**, 3722–3734.

Lauterborn,J.C. *et al.* (2021) Increased excitatory to inhibitory synaptic ratio in parietal cortex samples from individuals with Alzheimer’s disease. *Nat Commun*, **12**, 2603.

Logothetis,N.K. *et al.* (2001) Neurophysiological investigation of the basis of the fMRI signal. *Nature*, **412**, 150–157.

Maestú,F. *et al.* (2021) Neuronal Excitation/Inhibition imbalance: a core element of a translational perspective on Alzheimer pathophysiology. *Ageing Res Rev*, **69**, 101372.

Meijer,H.G.E. *et al.* (2015) Modeling focal epileptic activity in the Wilson-cowan model with depolarization block. *J Math Neurosci*, **5**, 7.

van Nifterick,A.M. *et al.* (2022) A multiscale brain network model links Alzheimer’s disease-mediated neuronal hyperactivity to large-scale oscillatory slowing. *Alzheimers Res Ther*, **14**, 101.

Obata,T. *et al.* (2004) Discrepancies between BOLD and flow dynamics in primary and supplementary motor areas : application of the balloon model to the interpretation of BOLD transients. **21**, 144–153.

Sanchez-Rodriguez,L.M., Khan,Ahmed F, *et al.* (2024) In-vivo neuronal dysfunction by Aβ and tau overlaps with brain-wide inflammatory mechanisms in Alzheimer's disease. *Front Aging Neurosci*, **16**.

Sanchez-Rodriguez,L.M., Khan,Ahmed F., *et al.* (2024) In-vivo neuronal dysfunction by Aβ and tau overlaps with brain-wide inflammatory mechanisms in Alzheimer's disease. *Front Aging Neurosci*, **16**.

Sanchez-Rodriguez,L.M., Bezgin,Gleb, *et al.* (2024a) Personalized whole-brain neural mass models reveal combined Aβ and tau hyperexcitable influences in Alzheimer's disease. *Commun Biol*, **7**, 528.

Sanchez-Rodriguez,L.M., Bezgin,Gleb, *et al.* (2024b) Personalized whole-brain neural mass models reveal combined Aβ and tau hyperexcitable influences in Alzheimer's disease. *Commun Biol*, **7**.

Simon,A.B. and Buxton,R.B. (2015) Understanding the dynamic relationship between cerebral blood flow and the BOLD signal: Implications for quantitative functional MRI. *Neuroimage*, **116**, 158–167.

Sotero,R.C. *et al.* (2009) Identification and comparison of stochastic metabolic/hemodynamic models (sMHM) for the generation of the BOLD signal. *J Comput Neurosci*, **26**, 251–69.

Sotero,R.C. and Trujillo-Barreto,N.J. (2008) Biophysical model for integrating neuronal activity, EEG, fMRI and metabolism. *Neuroimage*, **39**, 290–309.

Sotero,R.C. and Trujillo-Barreto,N.J. (2007) Modelling the role of excitatory and inhibitory neuronal activity in the generation of the BOLD signal. *Neuroimage*, **35**, 149–165.

Valdes-Sosa,P.A. *et al.* (2009) Model driven EEG/fMRI fusion of brain oscillations. *Hum Brain Mapp*, **30**, 2701–2721.

Wilson,H.R. and Cowan,J.D. (1972) Excitatory and inhibitory interactions in localized populations of model neurons. *Biophys J*, **12**, 1–24.
